# Right medial temporal lobe mass in a 25‐year‐old male

**DOI:** 10.1111/bpa.70093

**Published:** 2026-03-16

**Authors:** Jorge Samanamud, Cheyanne C. Slocum, Michael Christian Virata, Zied Abdullaev, Kenneth Aldape, Melissa Umphlett, Raymund L. Yong, Thomas P. Naidich, Nadejda M. Tsankova, Jamie M. Walker, Timothy E. Richardson

**Affiliations:** ^1^ Department of Pathology, Molecular and Cell‐Based Medicine Icahn School of Medicine at Mount Sinai New York New York USA; ^2^ Laboratory of Pathology National Cancer Institute, National Institutes of Health Bethesda Maryland USA; ^3^ Department of Pathology Mayo Clinic Rochester Minnesota USA; ^4^ Department of Neurosurgery Icahn School of Medicine at Mount Sinai New York New York USA; ^5^ Department of Diagnostic, Molecular and Interventional Radiology Icahn School of Medicine at Mount Sinai New York New York USA; ^6^ Nash Family Department of Neuroscience Icahn School of Medicine at Mount Sinai New York New York USA; ^7^ Friedman Brain Institute, Icahn School of Medicine at Mount Sinai New York New York USA

**Keywords:** diffuse glioneuronal tumor with oligodendroglioma‐like features and nuclear clusters (DGONC), high‐grade features, infiltrating glioma, methylation profiling

BOX 1 **Virtual glass slide.**

Access at https://isn‐slidearchive.org/?col=ISN&fol=Archive&file=BPA‐3472970.svs

## 1 CLINICAL HISTORY AND IMAGING

**FIGURE 1:**
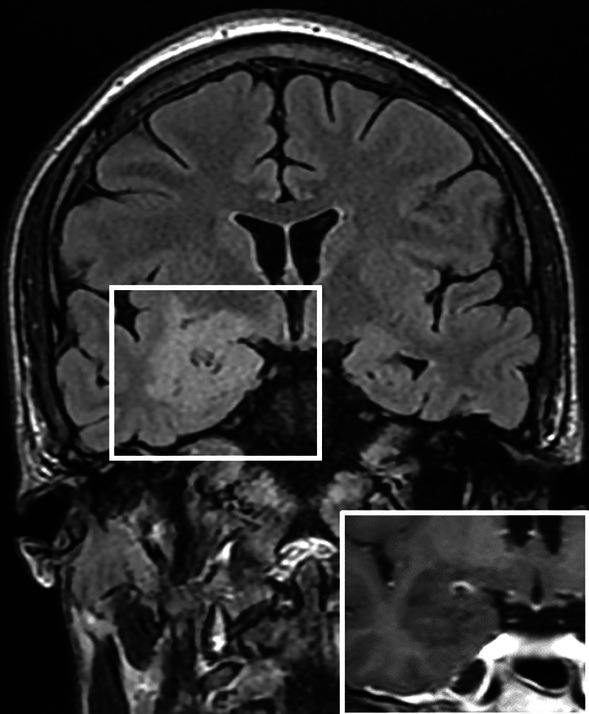
Coronal T2‐weighted FLAIR imaging demonstrates a hyperintense right medial temporal lobe mass that extends into the inferior right frontal lobe and bulges medially into the incisura. Gadolinium‐enhanced coronal T1‐weighted imaging shows the mass elevating the right middle cerebral artery but no significant contrast enhancement within the tumor (inset).

A 25‐year‐old male with no significant past medical, surgical, or neurologic history presented with a new‐onset seizure, which occurred while driving and led to a motor vehicle accident. His family history was notable for a paternal grandfather who died of glioblastoma at the age of 56. Brain magnetic resonance imaging (MRI) demonstrated a 3.4 × 3.2 × 3.1 cm expansile mass in the right medial temporal lobe with extension into adjacent basal forebrain structures. The lesion showed diffusion restriction, hyperintensity on T2‐Fluid‐Attenuated Inversion Recovery (T2‐FLAIR), and absence of significant contrast enhancement on T1‐weighted MRI (Figure 1). The patient subsequently underwent a right frontotemporal craniotomy with gross total resection confirmed by post‐contrast MRI and was discharged 2 days after surgery.

## 2 FINDINGS

**FIGURE 2:**
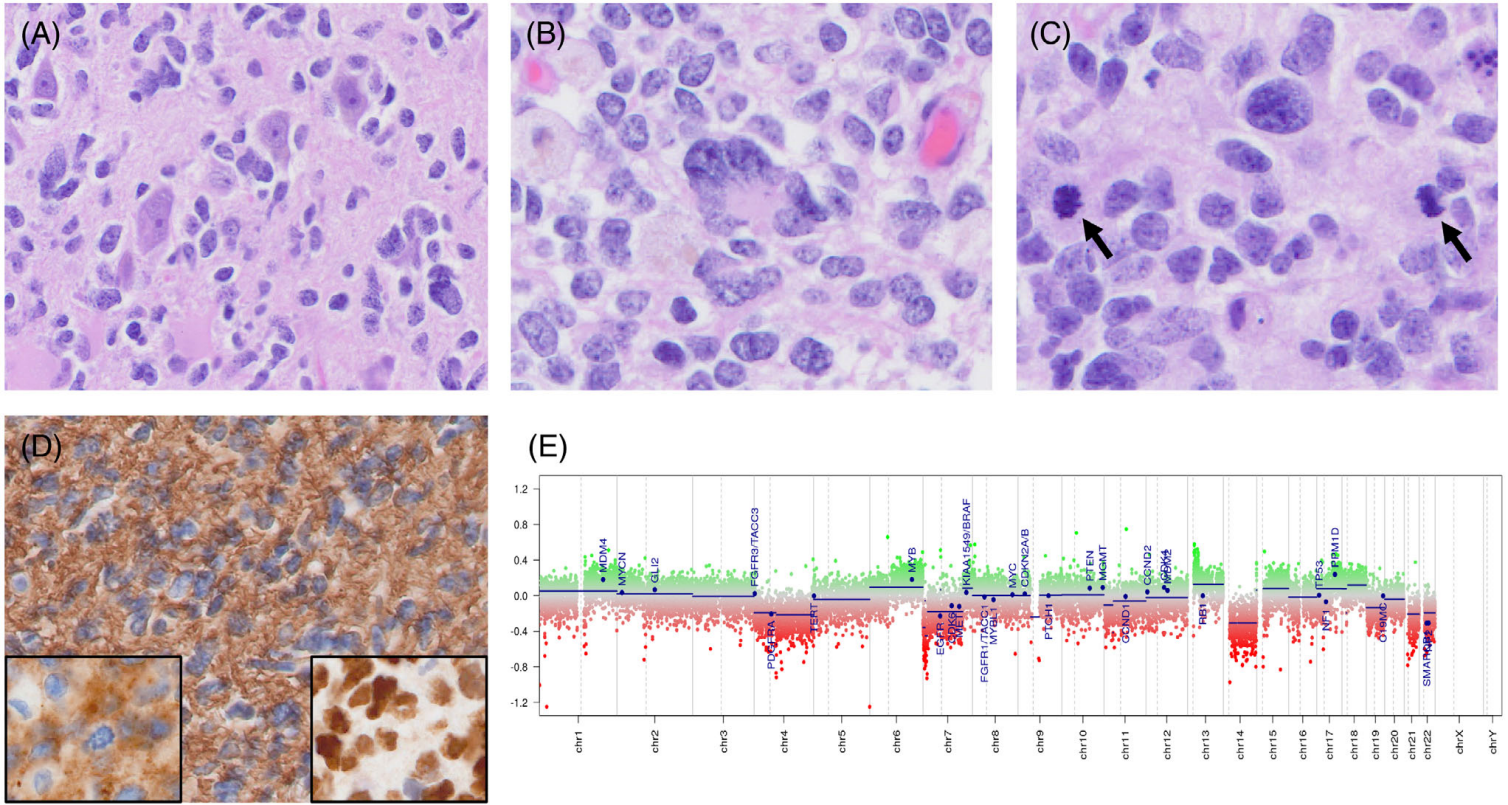
Hematoxylin & eosin (H&E) images demonstrate infiltration with perineuronal satellitosis (60×) (A), characteristic multinucleated cells (100×) (B), and frequent mitotic figures (arrows) (100×) (C). Immunohistochemistry demonstrates focally strong GFAP staining (40×), strong diffuse synaptophysin staining (left insert) (40×), and strong nuclear OLIG2 (right inset) (40×) (D). Copy number profiling reveals scattered losses, including characteristic chromosome 14 loss (E).

H&E‐stained sections demonstrated a diffusely infiltrating neoplasm composed of small to medium‐sized cells with scant cytoplasm and perinuclear halos (Box 1). There was perineuronal satellitosis (Figure 2A), scattered nuclear clusters and multinucleated cells (Figure 2B), microcalcifications, and focally frequent mitotic figures (up to 13/10 high power fields) (Figure 2C). Microvascular proliferation and necrosis were not identified. GFAP was focally strongly positive (Figure 2D). Synaptophysin was strongly and diffusely positive (Figure 2D [left insert]) and OLIG2 was strongly positive in tumor cell nuclei (Figure 2D [right insert]). Immunohistochemistry was negative for IDH1 R132H, H3K27M, and BRAFV600E. The Ki‐67 proliferation index was highly variable, ranging from less than 5% to over 30%. Fluorescent in situ hybridization (FISH) demonstrated retained 1p/19q and pyrosequencing demonstrated unmethylated *MGMT* promoter. Next‐generation sequencing revealed loss of chromosome 14, as well as two variants of uncertain significance, *FGFR1* p.E592G and *NOTCH1* p.L1805P, neither of which is predicted to be oncogenic (https://www.ncbi.nlm.nih.gov/clinvar/). Copy number profiling redemonstrated the loss of chromosome 14 along with other focal chromosomal losses (Figure 2E). DNA methylation profiling matched with high confidence (0.999 by Bethesda Classifier [v2] and >0.999 by Molecular Neuropathology classifier [v12b6]) to diffuse glioneuronal tumor with oligodendroglioma‐like features and nuclear clusters (DGONC).

## 3 DIAGNOSIS

Diffuse glioneuronal tumor with oligodendroglioma‐like features and nuclear clusters (DGONC), with high‐grade features.

## 4 DISCUSSION

Diffuse glioneuronal tumor with oligodendroglioma‐like features and nuclear clusters (DGONC) is a rare, methylation‐defined entity with only a limited number of cases reported in the literature to date. DGONC was initially recognized as a distinct DNA methylation cluster, then subsequently delineated as a histopathologically and molecularly distinct tumor type by Deng et al., who described its characteristic morphology and recurrent monosomy of chromosome 14 [1]. It is now listed in the 2021 WHO classification of CNS tumors as a provisional tumor type [2]. These neoplasms are composed of small to medium‐sized diffusely infiltrating cells with scant cytoplasm and frequent perinuclear halos, often accompanied by scattered multinucleated cells, characteristic nuclear clusters, and microcalcifications. These features may lead to an initial suspicion of oligodendroglioma, although they lack 1p/19q co‐deletion and *IDH1/2* mutation. Both low‐ and high‐grade features may be present, including microvascular proliferation, necrosis, and a variable mitotic rate. Approximately 45% of cases were originally diagnosed as WHO grade 4 tumors [1]. Immunohistochemically, they typically show OLIG2 and synaptophysin positivity and lack GFAP staining [2]. Monosomy 14 was described in 43/44 of reported cases along with other less‐frequent scattered copy number alterations (CNAs) [1, 3], although no recurrent mutations have been found [2]. The majority of cases occur in children, with a median age of 9 years; however, the age at diagnosis may vary widely (2–75 years). Limited clinical data suggest a 5‐year recurrence‐free and overall survival rates of 81% and 89%, respectively, even in cases with high‐grade features (https://neuro2.pathology.pitt.edu/dss/year/2022) [2].

Due to the limited number of reported cases, the full clinical, morphologic, immunophenotypic, and molecular spectrum of DGONC remains incompletely defined, and there is no established WHO grade. Although these tumors share a characteristic DNA methylation profile, emerging reports suggest meaningful biological heterogeneity within this group. Tauziède‐Espariat et al. described a methylation‐defined DGONC with distinct high‐grade features but lacking monosomy 14, instead having chromosome 17q gain and partial gain of 7q, underscoring potential variability in CNA patterns within this entity [3]. Other studies have highlighted consistently negative GFAP expression and relatively low Ki‐67 proliferative indices as common immunophenotypic features. The current case conforms to the prototypical DGONC morphologic and molecular profile, including oligodendroglioma‐like clear cell morphology, scattered multinucleated cells, microcalcifications, and monosomy 14, but demonstrates deviation in immunophenotype and proliferative behavior. Specifically, we observed strong GFAP expression, focally markedly elevated Ki‐67 index, and frequent mitotic activity, features that in another setting would raise concern for a high‐grade diffuse glioma. It is also notable that the current case has widely variable intratumoral proliferation indices. How such phenotypic and molecular variability will ultimately influence formal classification, grading, and clinical prognostication remains unknown and will require additional molecularly confirmed cases with long‐term outcome data.

## AUTHOR CONTRIBUTIONS

JS, CCS, MCV, and TER contributed to the study conception and design. JS, CCS, MCV, RLY, MU, NMT, JMW, and TER contributed materials, data collection, and performed pathologic analysis. ZA and KA performed methylation profiling. TPN performed radiologic analysis. All authors have reviewed and approved the final manuscript.

## FUNDING INFORMATION

No funding was received for this study.

## CONFLICT OF INTEREST STATEMENT

TER has received consulting fees from Servier Pharmaceuticals, which is unrelated to the current work. The results presented in this paper have not been published previously. The authors declare that they have no additional competing interests, conflicts of interest, or other relevant disclosures.

## Data Availability

The data that support the findings of this study are available from the corresponding author upon reasonable request.
